# New Evidence of the Importance of Weak Interactions in the Formation of PML-Bodies

**DOI:** 10.3390/ijms23031613

**Published:** 2022-01-30

**Authors:** Alexander V. Fonin, Sergey A. Silonov, Anna S. Fefilova, Olesya V. Stepanenko, Anastasia A. Gavrilova, Alexey V. Petukhov, Anna E. Romanovich, Anna L. Modina, Tatiana S. Zueva, Evgeniy M. Nedelyaev, Nadejda M. Pleskach, Mirya L. Kuranova, Irina M. Kuznetsova, Vladimir N. Uversky, Konstantin K. Turoverov

**Affiliations:** 1 Laboratory of Structural Dynamics, Stability and Folding of Proteins, Institute of Cytology, Russian Academy of Sciences, 4 Tikhoretsky Ave., 194064 St. Petersburg, Russia; silonovsa25@yandex.ru (S.A.S.); a.fefilova@incras.ru (A.S.F.); lvs@incras.ru (O.V.S.); asultanbekova@incras.ru (A.A.G.); anhel231097@mail.ru (A.L.M.); stellazueva@yandex.ru (T.S.Z.); nedelyaev99@mail.ru (E.M.N.); pleska@mail.ru (N.M.P.); mirya.san@yandex.ru (M.L.K.); imk@incras.ru (I.M.K.); 2 Almazov National Medical Research Centre, Institute of Hematology, 197341 St. Petersburg, Russia; petukhov_av@almazovcentre.ru; 3 Resource Center of Molecular and Cell Technologies, St-Petersburg State University Research Park, Universitetskaya Emb. 7–9, 199034 St. Petersburg, Russia; a.romanovich@spbu.ru; 4 Department of Molecular Medicine, Byrd Alzheimer’s Research Institute, Morsani College of Medicine, University of South Florida, Tampa, FL 33612, USA; vuversky@usf.edu

**Keywords:** membrane-less organelles (MLOs), PML-bodies, promyelocytic leukemia protein (PML) isoforms, acute hydrogen peroxide-induced oxidative stress, fluorescence recovery after photobleaching (FRAP), liquid–liquid phase separation (LLPS)

## Abstract

In this work, we performed a comparative study of the formation of PML bodies by full-length PML isoforms and their C-terminal domains in the presence and absence of endogenous PML. Based on the analysis of the distribution of intrinsic disorder predisposition in the amino acid sequences of PML isoforms, regions starting from the amino acid residue 395 (i.e., sequences encoded by exons 4–6) were assigned as the C-terminal domains of these proteins. We demonstrate that each of the full-sized nuclear isoforms of PML is capable of forming nuclear liquid-droplet compartments in the absence of other PML isoforms. These droplets possess dynamic characteristics of the exchange with the nucleoplasm close to those observed in the wild-type cells. Only the C-terminal domains of the PML-II and PML-V isoforms are able to be included in the composition of the endogenous PML bodies, while being partially distributed in the nucleoplasm. The bodies formed by the C-terminal domain of the PML-II isoform are dynamic liquid droplet compartments, regardless of the presence or absence of endogenous PML. The C-terminal domain of PML-V forms dynamic liquid droplet compartments in the knockout cells (PML^−/−^), but when the C-terminus of the PML-V isoform is inserted into the existing endogenous PML bodies, the molecules of this protein cease to exchange with the nucleoplasm. It was demonstrated that the K490R substitution, which disrupts the PML sumoylation, promotes diffuse distribution of the C-terminal domains of PML-II and PML-V isoforms in endogenous PML knockout HeLa cells, but not in the wild-type cells. These data indicate the ability of the C-terminal domains of the PML-II and PML-V isoforms to form dynamic liquid droplet-like compartments, regardless of the ordered N-terminal RBCC motifs of the PML. This indicates a significant role of the non-specific interactions between the mostly disordered C-terminal domains of PML isoforms for the initiation of liquid–liquid phase separation (LLPS) leading to the formation of PML bodies.

## 1. Introduction

At the beginning of the 21st century, ideas on the organization of the intracellular space underwent radical changes. It became obvious that liquid–liquid phase transitions (LLPTs) and liquid–liquid phase separation (LLPS) of biopolymers play a decisive role in structuring the intracellular space. The compartments (membrane-less organelles, MLOs) resulting from these phase transitions have all the characteristic properties of liquids. The main function of these compartments is the implementation in them of certain biochemical reactions specific for these organelles. Unlike organelles surrounded by a lipid membrane, the inclusion of target molecules in MLOs is carried out via multiple weak non-specific interactions, including electrostatic, cation-pi, and stacking interactions, which are determined by the charge of interacting macromolecules and the presence of aromatic groups in their composition [1,2,3,4,5]. Proteins that make up MLOs can also contain various motifs (SUMO, SIM, SH3-domains, proline-rich regions, etc.) that ensure their specific interaction with various partners. In the corresponding cases, intrinsically disordered proteins (IDPs) and RNA molecules; i.e., biopolymers that do not have an ordered structure, are able to separate into phases at relatively low concentrations due to their conformational heterogeneity and flexibility and the multivalency of their interactions [6,7,8].

These compartments include PML bodies, which are nuclear polyfunctional MLOs that are involved in the regulation of transcription, stress response, differentiation, and transition of cells to the senescent state [9,10,11,12]. The major component of these compartments is the promyelocytic leukemia (PML) protein. Due to alternative splicing, PML has at least seven main isoforms that differ in size and amino acid sequence of their C-terminal domains [13,14,15,16]. The N-terminal ordered region of the protein, which is the same in all PML isoforms, contains the so-called RBCC (Ring-Box-Coiled-Coil) motif consisting of several zinc-binding RING (Really Interesting New Gene) domains and two B-domains (B-Boxes), and also from the leucine-rich α-helical coiled-coil domain (there are 12 leucine residues in this region, which are L240, L247, L268, L278, L297, L298, L316, L319, L325, L332, L346, and L352) [10]. The C-terminus of most PML isoforms contains a nuclear localization signal (NLS) and a SUMO-interacting motif (SIM). SIM and RBCC provide the possibility of specific interaction of PML with a large number of partners, and NLS defines the nuclear localization of PML-bodies [10].

In view of the exceptional importance of PML bodies in normal cell life and in diseases [17], the study of PML bodies began long before the establishment of the determining role of LLPTs/LLPS in the formation of MLOs [18]. Since then, there has been an idea that the primary cause of the occurrence of PML bodies is the oxidative dimerization of PML molecules due to the formation of disulfide bonds between the ordered N-terminal domains of this protein [19,20,21,22,23,24]. Further oligomerization of PML is facilitated by specific interactions between the RBCC motifs of this protein’s molecules. The sumoylated proteins and SUMO-conjugating enzyme UBC-9 are attracted to the insoluble framework formed as a result of such interactions [12]. Sumoylation of PML at sites K65, K160, K226, and K490 mediated by UBC-9 allows regulation of PML–PML interactions and incorporation of client proteins into PML bodies using multivalent SUMO/SIM interactions [10]. Mature PML bodies have a toroidal topology [25]. From our point of view, the formation of disulfide bonds between PML molecules is possible only in the case of the appearance of preliminarily collected condensates, in which the concentration of PML molecules will become significantly (by orders of magnitude) higher compared to the concentration of PML molecules during their random distribution in the cell nucleus.

Our previous analysis of the sequences of PML isoforms revealed [26] that a number of PML isoforms, primarily PML-II, are prone to phase separation due to the polyampholytic properties and disordered nature of their C-terminal domains. We found the existence of a population of “small” PML bodies that do not have a pronounced toroidal structure, whose properties, according to FRAP analysis, are close to liquid-droplet compartments [26].

In this work, we characterized the role of disordered C-terminal domains of various PML isoforms in the formation of PML bodies and the response of these organelles to acute oxidative stress in the context of the LLPTs of PML isoforms, regardless of the ordered PML RBCC motifs.

## 2. Results and Discussion

In this work, we performed a comparative study of the formation of PML bodies by full-length PML isoforms and their C-terminal domains in the presence and absence of endogenous PML.

### 2.1. PML Bodies Formed by Exogenous PML I–V Isoforms in the Nuclei of PML Knockout HeLa Cells

**Figure 1:**
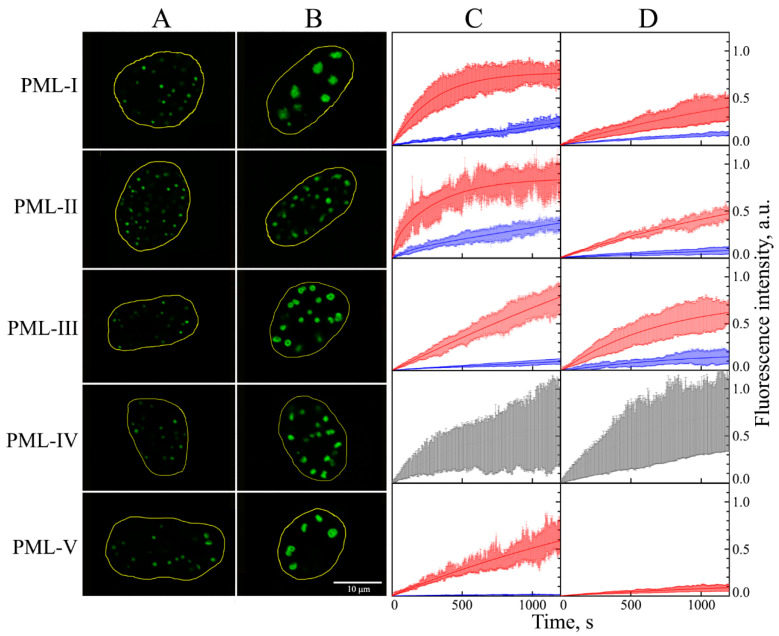
Localization, shape, and dynamic properties of PML bodies visualized by various exogenous EGFP-PML isoforms in the absence of all endogenous forms of PML protein in PML^−/−^ HeLa cells. Nuclear localization of normal, small (area < 1.5 μm^2^), and large (area > 1.75 μm^2^) PML-bodies with exogenous PML isoforms I–V are represented in Panel (**A**,**B**), respectively. Panel (**C**,**D**): Curves of EGFP fluorescence recovery after photobleaching of nuclear PML isoforms in PML-bodies in studied cells in absence of stress and in acute oxidative stress conditions, respectively. The curves of the photoreduction of PML isoforms in the composition of “small” PML bodies are shown in red, the curves of the photoreduction of PML isoforms in the composition of “large” PML bodies are shown in blue, and the curves of the photoreduction of PML-IV isoforms are shown in gray. Solid curves represent the fit of FRAP data in the framework of the mono-exponential approximation. The standard deviations of the data are shown by the error bars of the corresponding color.

In this work, we studied the dynamics of the exchange of PML-I–V isoforms with the nucleoplasm under the conditions of the transient expression of these proteins in endogenous PML knockout HeLa cells using the restoration of EGFP fluorescence after its photobleaching (FRAP). It was found that the PML-I–V nuclear isoforms are able to form compartments in the nuclei of PML^−/−^ HeLa cells with a morphology and size close to those of the endogenous PML-bodies in the absence of other PML isoforms (Figure 1). The exception is the PML-II isoform, whose exogenous expression in PML knockout HeLa cells leads to the formation of exclusively spherical bodies that do not have a toroidal structure, which is consistent with the data of [27].

The ability of individual PML-I–V isoforms to form condensates in the absence of endogenous PML forms in PML^−/−^ HeLa cells was shown in [27]. In this work, using super-resolution microscopy, the structure of such bodies and the effect of violation of the SIM motif in the sequence of various PML isoforms on the morphology of the condensates formed by them were established. PML-I, III, IV, and V isoforms have been shown to form bodies with a toroidal structure, the morphology of which is close to that of the endogenous PML bodies. The PML-II isoform, in the absence of other forms of PML, formed exclusively spherical bodies, in which the protein is distributed evenly throughout the volume. Disruption of the SIM motif in the sequence of PML-I and PML-IV isoforms resulted in the disruption of the toroidal topology of the bodies formed by them. In this work, we investigated the dynamic properties of the condensates formed by individual PML isoforms. 

The bodies formed by nuclear isoforms PML-I–V in the endogenous PML knockout HeLa cells are dynamic liquid droplet-like compartments (Figure 1). As well as in the wild-type cells, at least two populations of bodies can be distinguished among the compartmens formed by the individual PML isoforms: large bodies with a predominantly toroidal morphology and small spherical bodies, which differ in the dynamics of exchange of their PML proteins with the nucleoplasm [26]. The PML-IV isoform forms compartments in the endogenous PML knockout HeLa cells (Figure 1). The dynamics of these bodies gradually decreases with increasing the body size, which distinguishes such condensates from canonical PML bodies in wild-type cells and from structures formed by other PML isoforms in PML^−/−^ HeLa cells. The characteristics of the dynamics of the exchange of other PML isoforms that are part of the compartments formed by these proteins in PML^−/−^ HeLa cells correspond to the characteristics of the dynamics of the exchange of these PML isoforms with PML bodies in the wild-type cells [26]. It has been established (Figure 1) that the acute oxidative stress conditions arising from the treatment of PML^−/−^ HeLa cells with hydrogen peroxide affect the dynamics of the exchange of PML isoforms that are part of the condensates formed by these proteins within the nucleoplasm, as well as the dynamics of the exchange of the corresponding isoforms with endogenous PML-bodies in the wild-type cells [26]. The results obtained indicate that the exogenous PML isoforms form condensates with the same liquid droplet properties as the endogenous PML bodies. It should be noted that exogenous transient expression of PML isoforms in the presence of endogenous protein does not significantly affect the composition of PML bodies [28].

Accordingly, the dynamics of the exchange of PML-I–III and PML-V isoforms, which are part of the condensates, formed exclusively by these isoforms of the PML protein in the PML^−/−^ HeLa cells can be considered as a model for studying the dynamics of the exchange of these PML isoforms with the PML-bodies in the wild-type cells.

### 2.2. Insertion of C-Terminal Domains of PML Isoforms into the Endogenous PML Bodies

In a previous work, based on the analysis of the degree of disorder of the C-terminal residues [26], we made an assumption on the effects of the sequence encoded by the exons 4–6 of the *PML* gene and carrying the SIM motif on the inclusion of the C-terminal domains of PML-I–V in the endogenous PML-bodies. In this regard, the residues were chosen as C-termini are as follows: PML-I (residues 395–882), PML-II (residues 395–829), PML-III (residues 395–641), PML-IV (residues 395–633), PML-V (residues 395–611).

It is known that the assembly of PML bodies is critically dependent on the promyelocytic leukemia protein, an oncosuppressor encoded by the *PML* gene consisting of 10 exons [10]. The PML N-terminal RBCC domain consisting of the RING finger domain, two cysteine-rich B-boxes domains and coil–coil domain, which is the same for all PML isoforms (amino acid residues 1–394), is encoded by the first three exons of the gene of this protein, and, according to the results of the X-ray structural analysis [29,30], has an ordered structure. Variable C-terminal domains of PML with a disordered structure include amino acid sequences resulting from the alternating splicing of exons 4–9 of the gene of this protein [27]. Amino acid residues encoded by exons 4–6 (394–552), which are the same for PML nuclear isoforms, represent a rather disordered motif, but the involvement of this region of the amino acid sequence in PML formation has almost never been discussed. In a previous work, based on the analysis of the intrinsic disordered predisposition of PML, we proposed a new scheme for the domain organization of this protein, according to which the variable disordered C-terminal domains of PML include disordered regions of the amino acid sequence 394–552. This representation is not artificial, as one of the oncogenic forms of PML-RARα is formed as a result of the fusion of the first three exons of the *PML* gene and the gene for the retinoic acid receptor type alpha.

It was demonstrated that the C-terminal domains of the PML-II and PML-V isoforms, formed by amino acid residues 571–829 and 571–611 of the respective isoforms, respectively, can be incorporated into PML bodies in the wild-type cells, regardless of their RBCC and SIM motifs [31]. In work [27], it was found that the structure of bodies formed by PML-II, PML-III, and PML-V isoforms in the absence of endogenous PML practically does not depend on the presence of a SIM motif in the sequence of these proteins, which calls into question the decisive role of the SUMO/SIM interactions in the incorporation of these PML isoforms into PML bodies.

**Figure 2:**
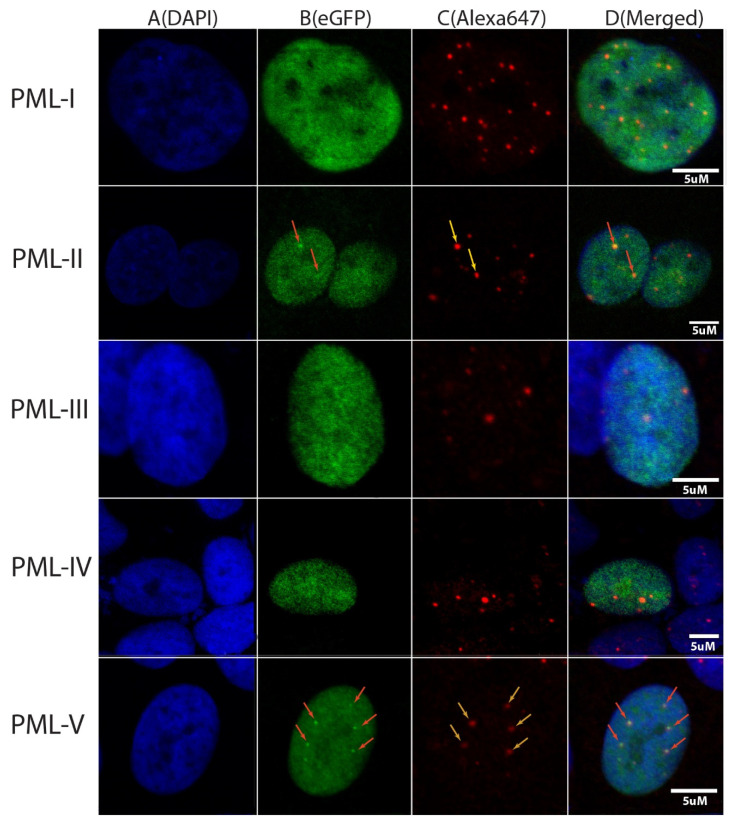
Colocalization of endogenous PML-bodies and C-terminal domains of PML isoforms in the HeLa cells using confocal fluorescent microscopy. Nucleus are colored by DAPI (column (**A**)). C-terminal domains of PML isoforms are visualized by expression of chimeric proteins EGFP fused with C-terminal domain of appropriate PML isoform (column (**B**)). The endogenous PML-bodies are visualized by cells staining with Anti-PML Alexa Fluor 647 antibodies (column (**C**)). Merging of images in columns (**A**–**C**) (column (**D**)).

In the present work, the colocalization of endogenous PML-bodies and C-terminal domains of PML isoforms in HeLa cells was investigated using confocal fluorescent microscopy. C-terminal localization was determined by the EGFP fluorescence, and endogenous PML-bodies are visualized by cell staining with Anti-PML Alexa Fluor 647 antibodies. The results of this analysis are shown in Figure 2. It turned out that under the conditions of exogenous transient expression of the C-terminal domains of the isoforms fused with the EGFP fluorescent protein PML-I (residues 395–882), PML-II (residues 395–829), PML-III (residues 395–641), PML-IV (residues 395–633) and PML-V (residues 395–611) only the C -terminal domains of the PML-II and PML-V isoforms are found in PML bodies in the wild-type HeLa cells (Figure 2, column 3), which is consistent with the data of [31]. The C-terminal domains of PML-II and PML-V are partially localized in the nucleoplasm. Therefore, the inclusion of sequences spanning the amino acid residues 394–552 into the PML C-terminal domains does not fundamentally affect the ability of the variable domains of the PML isoforms to interact with the PML bodies. These data also indicate that SUMO/SIM interactions alone are not sufficient for the incorporation of the PML isoforms into the PML bodies. In the light of our earlier analysis, these data may indicate that the disordered structure of their C-terminal domains may play a significant role in the inclusion of the PML-II and PML-V isoforms into the PML bodies.

### 2.3. The Mechanism of Incorporation of the PML-II Isoform into PML Bodies

In our previous work [26], we demonstrated that the PML-II isoform has the most disordered structure of the C-terminal domain and the greatest propensity for LLPS due to weak nonspecific interactions. The spherical shape of the bodies formed by the PML-II isoform (see Figure 1, in [27]) is also indirect evidence of the significant role of LLPS in the incorporation of the PML-II isoform into PML bodies.

**Figure 3:**
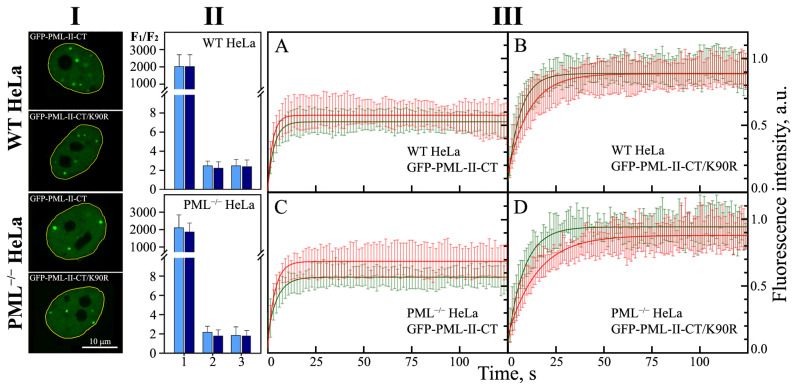
Localization, shape, and dynamic properties of the condensates formed by the C-terminal domain of PML-II isoform (PML-II-CT) and its mutant form with the K490R amino acid replacement (PML-II-CT/K490R) in wild-type HeLa cells (WT HeLa) and PML knockout HeLa cells (PML^−/−^ HeLa). Panel (**I**): Nuclear localization of condensates formed by PML-II-CT and PML-II-CT/K490R in the WTHeLa and PML^−/−^ HeLa. Panel (**II**): Relative contents of the isoform PML-II, PML-II-CT and PML-II-CT/K490R in bodies formed by these proteins and the nucleoplasm in the WT HeLa (upper panel), and the PML^−/−^ HeLa (lower panel) are shown by bars 1, 2 and 3, respectively. Light blue bars correspond to the normal conditions and that dark blue bars to H_2_O_2_-treated cells. The concentration of exogenously expressed forms of PML was estimated by the ratio of the average fluorescence intensity observed in the bodies (F1) to the average fluorescence intensity in a region of the same size in the nucleoplasm (F2). Bars and error bars represent means and s.d. (*n* = 3). Panel (**III**): Curves of EGFP fluorescence recovery after photobleaching of PML-II-CT (panels (**A**,**C**)) and PML-II-CT/K490R (panels (**B**,**D**)) in the WT HeLa (panels (**A**,**B**)) and the PML^−/−^ HeLa cells (panels (**C**,**D**)). The curves of the photoreduction of the target proteins at the normal conditions are shown in red, and the stress conditions (H_2_O_2_) are shown in green. Solid curves represent the fit of the FRAP data in the framework of the mono-exponential approximation. The standard deviations of the data are shown by the error bars of the corresponding color.

When PML bodies are formed by the full-length PML isoforms, the vast majority of PML molecules are concentrated in PML bodies. Deletion of the RBCC motif (PML-II-CT) causes an increase in the concentration of PML-II in the nucleoplasm by almost three orders of magnitude, regardless of the presence of the endogenous PML in cells (Figure 3, Panel II). This distribution of the PML-II C-terminal domain between the nucleoplasm and the PML-II-CT condensates formed is apparently a consequence of the phase separation under these conditions.

We studied the dynamic characteristics of the condensates formed by the C-terminal domain of the PML-II isoform in the absence and presence of endogenous PML; i.e., in the knockout (PML−/− HeLa) and the wild-type (WT HeLa) cells. The characteristics of the exchange of the C-terminal domain of PML-II within an endogenous PML-body and in condensates formed by this protein with the nucleoplasm are close to each other and differ significantly from the characteristics of the exchange of the full-sized PML-II under the studied conditions.

Both in the wild-type and HeLa cells knockout for the endogenous PML, the FRAP curves of the EGFP-C-terminal PML-II chimeric proteins, which are a part of the PML bodies, indicate a rapid but incomplete restoration of the EGFP fluorescence after its photobleaching (Figure 3 Panels III (A,C)). In addition, the dynamics of the exchange of the C-terminus of PML-II, which is a part of the endogenous PML-bodies with the nucleoplasm, does not depend on the size of these organelles, unlike the PML-II full-length isoform [26]. The high rate of the exchange of the contents of the condensate with the surrounding space is a characteristic feature of the structures formed as a result of LLPS.

It is known that the LLPS can be initiated by the SUMO/SIM interactions. The PML-II C-terminal domain contains the Lys490 sumoylation site. Disruption of the sumoylation of the studied protein by replacing K490R allowed us to determine the contribution of the SUMO/SIM interactions to the formation of condensates by the C-terminal domain of PML-II. The introduction of the K490R substitution into the sequence of the C-terminal domain of PML-II slightly slowed down the rate of the exchange of the molecules of the studied protein with the nucleoplasm, which led to the almost complete absence of the immobile fraction in the condensates formed by the PML-II C-terminal domain, both in the endogenous PML knockout cells and in the wild-type HeLa cells. Therefore, the K490R amino acid substitution promotes the formation of condensates by the C-terminal domain of PML-II with fully liquid droplet properties (Figure 3, Panel III (B,D)).

The role of sumoylation of the PML C-terminal domain at the K490 site in the condensate formation by this domain was also studied under the conditions of acute oxidative stress caused by the treatment of the studied cells with high concentrations of hydrogen peroxide. It is known that the acetylation of the PML sequence at 487 lysine residue and sumoylation at 490 lysine residue are mutually exclusive [32]. At the same time, high concentrations of reactive oxygen species created by H_2_O_2_ promote sumoylation at residue 490 and deacetylation at residue 487 [32]. Accordingly, the treatment of the studied cells with H_2_O_2_ had a multidirectional effect on the dynamic characteristics of the condensates formed by the wild-type C-terminal domain of PML-II and its mutant form with the K490R substitution (Figure 3, Panel III (B,D)). Acute oxidative stress caused a slight decrease in the rate of EGFP fluorescence recovery and a decrease in the proportion of the mobile fraction in the composition of the condensates formed by the wild-type PML-II C-terminal domain. Apparently, these changes are due to the increase in the protein sumoylation at the 490 lysine residue.

An increase in the exchange rate of the mutant form of the PML-II C-terminal domain with the K490R substitution, which is part of the condensates of this protein with the nucleoplasm, is apparently associated with the deacetylation of K487. As is known, acetylation of protein sequences can weaken their phase separation due to the neutralization of the positive charge of lysine [33].

The data obtained indicate the ability of the C-terminal domain of PML-II to separate into phases in the intranuclear space, regardless of the strong interactions caused by the oligomerization of PML RBCC motifs. Condensation of the C-terminal domain of PML-II into liquid droplets is due to two factors: SUMO/SIM interactions and weak non-specific interactions due to the disordered structure of the C-terminal domain of PML-II.

### 2.4. Mechanism of Incorporation of the PML-V Isoform into PML Bodies

**Figure 4:**
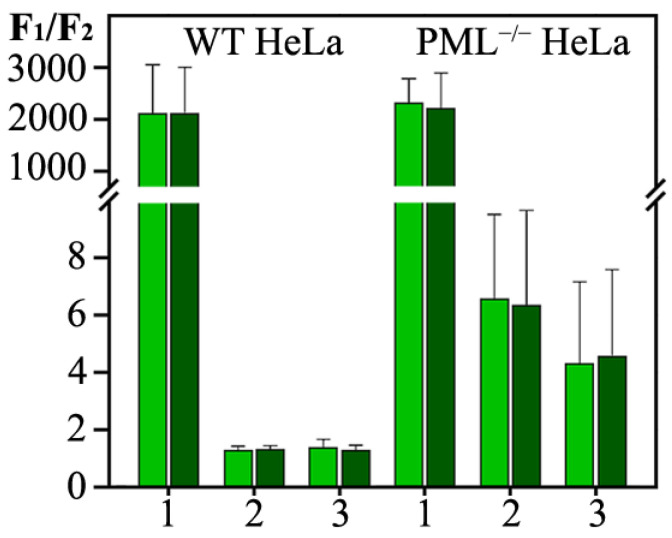
Relative contents of the isoform PML-V, PML-V-CT and PML-V-CT/K490R in bodies formed by these proteins and nucleoplasm in the WT HeLa (on the left), and in the PML^−/−^ HeLa (on the right) are presented by bars 1, 2, and 3, respectively. Light green bars correspond to the normal conditions and that dark green bar correspond to the H_2_O_2_-treated cells. The concentration of exogenously expressed forms of PML was estimated by the ratio of the average fluorescence intensity observed in the bodies to the average fluorescence intensity in a region of the same size in the nucleoplasm. Bars and error bars represent means and s.d. (*n* = 3).

The PML-V form does not have the same high degree of intrinsic structure disorder as PML-II. However, similar to the C-terminal domain of PML-II, the C-terminal domain of PML-V can be incorporated into the endogenous PML bodies and form condensates in the endogenous PML knockout cells. As with the PML-II isoform, deletion of the N-terminal domain of the PML-V causes a partially diffuse distribution of this protein in the nucleoplasm (Figure 4).

The dynamic characteristics of the exchange of the C-terminal domain of PML-V with the endogenous PML bodies differ radically from the characteristics of the exchange of the full-length protein with these organelles. The C-terminal domain of PML-V, which is a part of the endogenous PML-bodies, practically does not exchange with the nucleoplasm in the wild-type cells (data not shown). This indicates strong interactions between the C-terminus of PML-V and components of PML bodies. Previously, a hidden α-helix [31] was identified in the C-terminal domain of PML-V, apparently responsible for this effect. It is possible that this α-helix is involved in the interaction with the coiled-coil motifs of the N-terminal domains of the PML isoforms that are part of the PML bodies.

**Figure 5:**
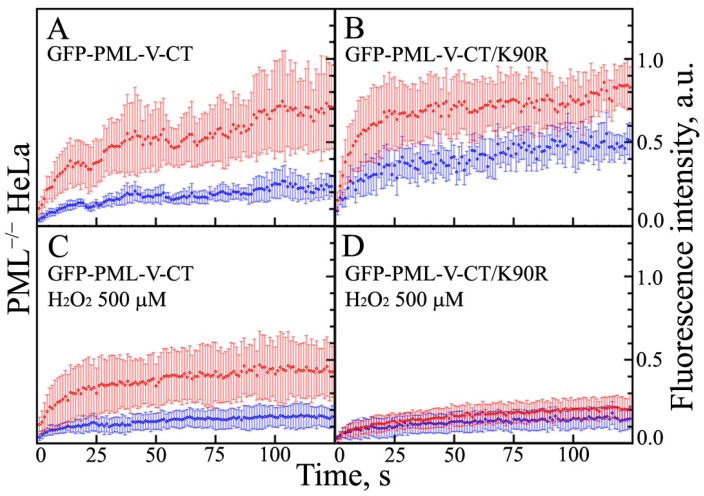
Dynamic properties of the condensates formed by the C-terminal domain of PML-V isoform and its mutant form with the K490R amino acid replacement in PML−/− HeLa cells. EGFP fluorescence recovery after photobleaching of C-terminal domain of PML-V isoform. Panels (**A**,**C**) present WT C-terminal domain of PMLV isoform and Panels (**B**,**D**) presents its mutant form with amino acid replacement K490R in normal (Panels (**A**,**B**)) and stressed (Panels (**C**,**D**)) conditions. The curves of the photoreduction of PML isoforms in the composition of “small” and “large” PML bodies are shown in red and blue. Solid curves represent the fit of the FRAP data in the framework of the mono-exponential approximation. The standard deviations of the data are shown by error bars of the corresponding color.

In the absence of endogenous PML, the C-terminal domain of the PML-V isoform is capable, just as the full-length isoform, of forming the two populations of condensates of different sizes with fast and slow dynamics of exchange with the nucleoplasm (Figure 5). The FRAP curves indicate a significant proportion of immobile molecules in all compartments formed by the C-terminal domain of the PML-V isoform. The introduction of the K490R substitution into the PML-V amino acid sequence, which disrupts the sumoylation of this protein, increases the dynamics of the exchange of this protein with the nucleoplasm and increases the proportion of this protein molecules localized in the nucleoplasm. This indicates a significant role of SUMO/SIM interactions in the formation of condensates by the PML-V C-terminal domain in the nucleoplasm of endogenous PML knockout HeLa cells. 

Treatment of the studied cells with high concentrations of hydrogen peroxide causes a significant decrease in the dynamics of the exchange of the C-terminal domain of PML-V and its mutant form with the K490R replacement, which are a part of the condensates formed by them, with the nucleoplasm. At the same time, the dynamics of exchange of the mutant form of K490R under the conditions of the acute oxidative stress slows down significantly more than that of the wild-type domain. It is known that the C-terminal domain of PML-V carries a significant negative charge. It is possible that the appearance of a positive charge cluster in the sequence of the PML-V C-terminal domain due to the K490R substitution and deacetylation at K487 under the conditions of acute oxidative stress contributes to the formation of non-dynamic condensates of this protein due to the enhanced electrostatic interactions.

The data obtained allow us to conclude that weak nonspecific interactions play a somewhat less significant role in the formation of condensates by the C-terminal domain of PML-V than in the formation of the condensates by the C-terminal domains of the PML-II isoform.

## 3. Materials and Methods 

### 3.1. Plasmids

On the basis of the pEGFP-C1/3 vectors, plasmids carrying genes encoding fusion proteins of 5 PML isoforms and their C-terminal domains with green fluorescent protein (GFP) were developed and created. The sequences of PML isoforms according to the UniProt P-29590 database were used as reference sequences. The PML-I isoform was kindly provided by Prof. Roger Everett (UK). On the basis of this construct, plasmids were created carrying the genes encoding the remaining PML isoforms. The resulting constructs were supplied by the PCR with Q5 polymerase (NEB, Ipswich, MA, USA) with the corresponding primers. PCR products were precipitated with ethyl alcohol with ammonium acetate, dissolved in water, and treated with restriction enzymes. Restriction products were purified from agarose gel with Cleanup Mini kit (Evrogen, Moscow, Russia) and ligated to each other with T4 ligase (Thermo Fisher Scientific, Waltham, MA, USA). 

The electroporation of *Escherichia coli* DH5-α cells was performed with the resulting ligase mixture. Several clones were selected for sequencing. Isolation of plasmids was carried out with the Plasmid Miniprep kit (Evrogen, Moscow, Russia). Next, the inserts of the obtained plasmids were sequenced with the preparation of samples using the BigDye Terminator v3.1 Cycle Sequencing Kit (Thermo Fisher Scientific, Waltham, MA, USA) and their subsequent analysis using the ABI PRISM 3500 (Applied Biosystems, Foster City, CA, USA). Clones in which the translated gene sequence coincides with the reference were selected. The transfection of the developed constructs into HeLa cells was carried out using the Effectene agent according to the manufacturer’s instructions.

### 3.2. Live Cell Imaging

HeLa cells were cultured in DMEM (GIBCO, Gaithersburg, MD, USA) medium containing 10% FBS (Thermo Fisher, Waltham, MA, USA), penicillin, streptomycin, and glutamate (Thermo Fisher, Waltham, MA, USA) at 37 °C with 5% CO_2_ in a humidified incubator. 

Cell imaging was performed by planting HeLa cells on pre-treated with poly-L-lysine (Sigma–Aldrich, St. Louis, MO, USA) 35 mm glass Ibidy and Eppendorf plates. The constructs were visualized by irradiating the cells with a laser with a wavelength of 488 nm to excite EGFP fluorescence. Visualization of the cells nuclei of was performed using a fluorescent dye DAPI by irradiating the cells with a laser with a wavelength of 405 nm to excite dye fluorescence. Resulting fluorescence was recorded using an Olympus FV3000 confocal microscope (60 × Oil immersion objective, NA 1.42). Images were corrected for background signal and high-frequency noise using ImageJ software (version 1.53c).

### 3.3. Fixed Cell Imaging

HeLa cells were cultivated on poly-L-lysine-coated microscopy glass coverslips and transfected with 2.5 μg of plasmid carrying various PML isoforms conjugated to eGFP 48 h before fixation. First, the cells were washed twice with phosphate-buffered saline (PBS) and fixed with 4% paraformaldehyde (PFA) (Sigma–Aldrich, St. Louis, MO, USA) at room temperature (RT) for 20 min. Then, the cells were washed with PBS twice for 10 min and permeabilized for 10 min with 0.5% Triton X-100 in PBS. Then, the cells were washed with PBS for 10 min, followed by blocking for 1h at RT with 3% BSA (Sigma–Aldrich, St. Louis, MO, USA) solution in PBS. Then, cells were placed in the hybridization chamber and stained with 1:100 Anti-PML Alexa Fluor647 Antibody (sc-966 AF647, Santa Cruz Biotechnology, Dallas, TX, USA) at 4 °C overnight. After that, the cells were washed twice with PBS at RT for 10 min. Thereafter, the cells were stained with 4′,6-diamidino-2-phenylindole (DAPI) (Thermo Fisher Scientific, Waltham, MA, USA) (300 nM in DMF) for 2 min, washed with PBS, and studied by confocal microscopy.

### 3.4. Fluorescence Recovery after Photobleaching (FRAP)

The analysis of the dynamics of PML isoforms in PML-bodies was carried out by restoring the EGFP fluorescence after photobleaching according to the following scheme: obtaining three images of the studied object before its photobleaching, photobleaching of target structures by irradiating them with a 488 nm laser with a power of 10 mW for 5 s, and obtaining an image photo restoration of target objects for 20 min. FRAP curves were normalized on post-bleach fluorescence intensity. Images were analyzed using ImageJ software. EGFP fluorescence photoreduction curves for various PML isoforms were characterized within the mono-exponential approximation and the half-recovery time of EGFP fluorescence; the proportion of mobile and immobile fractions for each PML isoform were determined. Curves were analyzed using GraphPad Prism software.

## 4. Conclusions

The data obtained indicate that the C-terminal domains of the PML-II and PML-V isoforms are not only able to be incorporated into the endogenous PML bodies, but also can form dynamic liquid-droplet compartments in HeLa PML−/− cells, while partially passing into the nucleoplasm. At the same time, the morphology and dynamic properties of the compartments formed with the participation of the C-terminus of the PML-II isoform in PML−/− and wild-type cells practically do not differ, which cannot be said about the characteristics of the compartments formed by the PML-V. It has been demonstrated that the K490R substitution, which disrupts PML sumoylation, promotes an increase in the diffuse distribution of the C-terminal domains of the PML-II and PML-V isoforms in the HeLa cells knockout for the endogenous PML, but not in the wild-type cells. At the same time, the analysis of the dynamic characteristics of the compartments, which include the C-terminal domains of the PML-II/K490R and PML-V/K490R isoforms, demonstrated that a distortion of the sumoylation causes the almost complete disappearance of the immobile fraction of these proteins in the bodies formed by them. The course of the FRAP curves of the analyzed proteins during the treatment of the studied cells with hydrogen peroxide also indicates a significant effect of the sumoylation of the C-terminal domains of PML-II and PML-V on the characteristics of the condensates formed by these proteins.

Taken together, the data obtained confirmed our earlier hypothesis [24] on the significant role of weak nonspecific interactions mediated by disordered C-terminal domains of PML isoforms in the formation of PML bodies.

## Data Availability

Not applicable.
